# Transcriptional implications of intragenic DNA methylation in the oestrogen receptor alpha gene in breast cancer cells and tissues

**DOI:** 10.1186/s12885-015-1335-5

**Published:** 2015-05-01

**Authors:** Natalie S Shenker, Kirsty J Flower, Charlotte S Wilhelm-Benartzi, Wei Dai, Emma Bell, Edmund Gore, Mona El Bahrawy, Gillian Weaver, Robert Brown, James M Flanagan

**Affiliations:** 1 Department of Surgery and Cancer, Epigenetics Unit, Division of Cancer, Faculty of Medicine, Imperial College London, 4th Floor IRDB, Hammersmith Campus, Du Cane Road, London, W12 0NN UK; 2 Current Address: The University of Hong Kong, Pokfulam, Hong Kong, P. R. China; 3 Queen Charlotte and Chelsea Hospital Milk Bank, Du Cane Road, London, UK

**Keywords:** Intragenic, DNA methylation, Breast cancer, ESR1, Breast epithelial cells, Breast cancer campaign tissue bank, Breast milk

## Abstract

**Background:**

DNA methylation variability regions (MVRs) across the oestrogen receptor alpha (*ESR1*) gene have been identified in peripheral blood cells from breast cancer patients and healthy individuals. In contrast to promoter methylation, gene body methylation may be important in maintaining active transcription. This study aimed to assess MVRs in *ESR1* in breast cancer cell lines, tumour biopsies and exfoliated epithelial cells from expressed breast milk (EBM), to determine their significance for *ESR1* transcription.

**Methods:**

DNA methylation levels in eight MVRs across *ESR1* were assessed by pyrosequencing bisulphite-converted DNA from three oestrogen receptor (ER)-positive and three ER-negative breast cancer cell lines. DNA methylation and expression were assessed following treatment with DAC (1 μM), or DMSO (controls). *ESR1* methylation levels were also assayed in DNA from 155 invasive ductal carcinoma biopsies provided by the Breast Cancer Campaign Tissue Bank, and validated with DNA methylation profiles from the TCGA breast tumours (n = 356 ER-pos, n = 109 ER-neg). DNA methylation was profiled in exfoliated breast epithelial cells from EBM using the Illumina 450 K (n = 36) and pyrosequencing in a further 53 donor samples. *ESR1* mRNA levels were measured by qRT-PCR.

**Results:**

We show that ER-positive cell lines had unmethylated *ESR1* promoter regions and highly methylated intragenic regions (median, 80.45%) while ER-negative cells had methylated promoters and lower intragenic methylation levels (median, 38.62%). DAC treatment increased ESR1 expression in ER-negative cells, but significantly reduced methylation and expression of ESR1 in ER-positive cells. The *ESR1* promoter was unmethylated in breast tumour biopsies with high levels of intragenic methylation, independent of ER status. However, *ESR1* methylation in the strongly ER-positive EBM DNA samples were very similar to ER-positive tumour cell lines.

**Conclusion:**

DAC treatment inhibited *ESR1* transcription in cells with an unmethylated *ESR1* promoter and reduced intragenic DNA methylation. Intragenic methylation levels correlated with ESR1 expression in homogenous cell populations (cell lines and exfoliated primary breast epithelial cells), but not in heterogeneous tumour biopsies, highlighting the significant differences between the *in vivo* tumour microenvironment and individual homogenous cell types. These findings emphasise the need for care when choosing material for epigenetic research and highlights the presence of aberrant intragenic methylation levels in tumour tissue.

**Electronic supplementary material:**

The online version of this article (doi:10.1186/s12885-015-1335-5) contains supplementary material, which is available to authorized users.

## Background

Breast cancer is the leading cause of cancer in women, and its incidence continues to rise, particularly in developed countries [1]. Strong evidence exists to support the role of aberrant epigenetic mechanisms in breast tumorigenesis, of which the most intensively investigated are changes in DNA methylation [2-4]. DNA methylation is evolutionarily the oldest and perhaps best studied mechanism of epigenetic transcriptional regulation, whereby a methyl group is covalently added to the 5-carbon of cytosine bases in a cytosine-guanine dinucleotide (CpG site). CpG sites tend to cluster into non-random CpG islands (CGIs) around the transcription start sites (TSS) of approximately 60% of genes. Dogma states that methylation of the promoter region-associated CGIs leads to conformational changes in the DNA strand and regional chromatin [5,6], which inhibit the initiation of the transcriptional machinery and prevent the recruitment of RNA polymerase II. If the CGI is unmethylated, the gene should be actively transcribed.

A recent review indicated that the differential methylation of intragenic variable regions may have important implications for transcription and cell-specific differentiation [7]. Changes in intragenic methylation (IGM) levels may represent the consequences of the transcriptional machinery [8], or a functionally relevant mechanism that affects transcriptional efficiency or gene stability [9-11]. It is likely that there are gene-to-gene subtleties in such mechanisms, and functionally important genes in breast cancer therefore warrant closer investigation as the transcriptional regulation of genes during breast tumorigenesis and throughout the disease course remains poorly understood. One such gene, oestrogen receptor alpha (*ESR1*), is of crucial importance in terms of both diagnostic and prognostic implications in breast cancer [12-14]. A previous study from our group indicated that regions of DNA methylation variability (MVRs) exist across the *ESR1* gene in peripheral blood cells from breast cancer patients compared to healthy matched controls [15], but the functional implications of this variability remains unknown.

Based on the hypothesis that IGM may play an important role in transcription [16-19], we aimed to ascertain whether IGM patterns differed in human breast cancer cells lines that were positive (n = 3) or negative (n = 3) for ESR1 expression. We also explored the effects on the cells in terms of the methylation and transcription of *ESR1* after treatment with a demethylating agent, decitabine (DAC), Furthermore, methylation levels across the *ESR1* gene were assessed in 155 samples of human breast cancer, and in 89 samples of exfoliated breast epithelial cells from donated expressed breast milk (EBM) from healthy women.

## Methods

### Cell lines

Six cell lines were obtained from stocks at the Hammersmith Hospital or purchased (ATCC, VA, USA). Of these, three were confirmed as ESR1-positive (T47D, MCF7, and BT474) and three were ESR1-negative (MDA-MB-231, BT549, and SKBR3), verified by STR profiling. Cells were cultured in sterile conditions at 37°C in a humidified atmosphere with 5% carbon dioxide, and maintained in either DMEM (Sigma-Aldrich, Poole, UK) or RPMI (Sigma) supplemented with 10% fetal calf serum (FCS; Sigma) and 5 ml L-glutamine. Cells were passaged when their confluence exceeded 70%.

### Decitabine treatment

The effect of increasing concentrations of DAC on the six cell lines was assessed using the MTT (3-(4,5-dimethylthiazol-2-yl)-2,5-diphenyltetrazolium bromide) dye reduction assay. Decitabine (DAC; Sigma-Aldrich) was re-suspended in 2.2 ml 100% dimethyl sulphoxide (DMSO; Sigma-Aldrich), and made up to 0.5, 1, 5, 10, or 20 μM compared to growth medium (0 μM) alone as the negative control. Assays were performed in triplicate, and the MTT assay was performed using 20 μl CellTiter 96 Aqueous One Solution Cell Proliferation Assay (Promega, Madison, WI, USA) according to the manufacturer’s protocol. The results indicated that cell viability was preserved for each cell line at ≤5 μm DAC. Therefore, 1 μm DAC was chosen for the subsequent cell culture experiments to prevent DAC cytotoxicity.

Fresh aliquots of DAC and DMSO were used for each experiment. Each cell line was cultured in 75 cm^3^ flasks in 10 ml DMEM + 10% FCS with 1 μM DAC or DMSO for 7 d in triplicate, and at three separate time points. After the appropriate duration of incubation, cells were trypsinised and counted. Cell pellets were collected after three PBS washes and centrifugation at 1,500 rpm for 5 min, and divided in half for DNA and RNA extraction. DNA was extracted using the QIAamp® DNA Mini Kit (Qiagen, Crawley, UK), and concentration and quality was assessed using a Nanodrop1000 spectrophotometer (ThermoScientific, UK). DNA was stored at −20°C until bisulphite conversion.

### Methylation analyses

Bisulphite conversion changes all unmethylated cytosine bases into uracil, therefore allowing the identification of unconverted cytosines as those that are methylated by pyrosequencing [20]. DNA samples were bisulphite-converted using the EpiTect kit according to the manufacturer’s protocol (Qiagen). Bisulphite-treated DNA was then desulphonated, washed and eluted prior to its use in PCR.

PCR assays were designed using a semi-nested approach to avoid the amplification of repetitive elements, such as long-interspersed nuclear elements (LINE) segments, which are often present in the MVRs across *ESR1* [15]. A biotinylated tag was placed on one of the primers, and a common biotinylated primer was used for all reactions as described in previous reports [15,21]. The list of PCR and sequencing primer sequences is given in Additional file 1: Table S1. The prepromoter region assayed was found between −4839 and −3904 bp upstream of the transcription start site (TSS), while the promoter region assayed comprised CpG sites from the TSS to 178 bp into the gene. Reactions took place in a thermal cycler under the following conditions: incubation at 95°C for 10 min; an initial 20 sec incubation at 95°C followed by 10 cycles of a 20 s incubation at 60°C (temperature decreased by 1.0°C every cycle) and incubation at 72°C for 20 s; second round PCR steps were performed using nested primers as follows: 30 cycles at 95°C for 20 s, 50°C for 20 s and 72°C for 20 s followed by a final incubation of 72°C for 5 min, with the exception of MVR 7b which only required a single-step PCR amplification. Products were assessed for quality by agarose gel electrophoresis and stored at 4°C until pyrosequencing.

Bisulphite-converted DNA samples were pyrosequenced using specific sequencing primers designed with the use of the PyroQ assay design software (Pyromark MD, Qiagen), and assay were performed on a Pyromark MD pyrosequencer using standard protocols and controls. Assays were repeated if any the inbuilt quality control measures were flagged.

### RNA isolation, cDNA synthesis and qRT-PCR assays of cell line RNA

RNA was isolated from cell pellets using the Qiagen RNeasy® Mini kit (Qiagen), according to the manufacturer’s instructions. The concentration of each RNA sample was assessed with the Nanodrop and all OD_260/280_ ratios were >1.8. cDNA was synthesised from 2 μg of each RNA sample using the SuperScript™ III First Strand Synthesis System for RT-PCR (Invitrogen, Carlsbad, CA, USA). Negative controls were prepared without Superscript™ III RT for each group of samples. All samples were stored at −20°C prior to RT-PCR.

Each qRT-PCR analysis was performed in triplicate for each of the duplicate experimental sets of cDNA from the six cell lines. Each qRT-PCR run was performed in duplicate using primers that were specific for *ESR1* mRNA and for the housekeeping gene, *GADPH* (forward, 5′-TCCCATCACCATCTTCCA-3′ and reverse, 5′-CATCACGCCACAGTTTCC-3′) [22]. The details of primers used are given in Additional file 1: Table S2, and assays were checked using gel electrophoresis to confirm the expected amplicon sizes were valid. All primers were 100% specific for the region of interest. The plate was centrifuged briefly and placed in a C1000™ Thermal Cycler (BioRad, UK). The PCR conditions were established using the Bio-Rad CFX Manager software as follows: 95°C for 3 min denaturing step; 42 cycles of 10 s at 95°C, 10 s at 56°C and 30 s at 72°C; 10 s at 95°C and a melt curve cycle of 5 min that ranged from 72°C to 95°C. Cycle threshold (Ct) values were recorded at a logarithmic threshold of 10^3^, and the relative quantitative expression of *ESR1* mRNA in each sample was calculated by the ^-∆∆^Ct conversion.

### Breast tumour samples

Power calculations based on the observed differences in cell lines suggested that group sample sizes of n = 45 would be sufficient to reach >90% power at alpha = 0.01 to detect the maximum difference observed (p6), and >80% power at alpha = 0.05 to detect a significant difference of >40% methylation (observed at other sites across ESR1). We received samples from the Breast Cancer Campaign Tissue Bank, comprising 10 formalin-fixed paraformaldehyde slides per tumour for 135 tumours (45 ER-negative tumours samples, 45 ER-positive grade 2 tumours and 45 ER-positive grade 3 tumours, as defined from histopathological review by MEB). Furthermore, we received 20 samples of fresh frozen (FF) tumours matched to FFPE samples for quality control purposes. This study was approved by the Ethics Committee of the Breast Cancer Campaign Tissue Bank (Approval no. BCC-TB00001). All H&E stained slides were reviewed by a pathologist to define the percentage of tumour with a minimum cut-off of >70%.

Slides were dewaxed for 10 min in Histoclear, followed by 10 min in 100% ethanol and another 10 min in fresh 100% ethanol. Slides were prepared with Levi buffer using standard techniques, and DNA was extracted using the phenol:chloroform technique. DNA concentrations and quality were assessed by the Nanodrop. Bisulphite conversions and pyrosequencing analyses were performed as described above. For the DNA extracted from FFPE slides, different primers had to be described with amplicons of <120 bp owing to the relative fragmentation of the DNA after formalin treatment, as DNA quality was poorer in these 135 samples (Additional file 1: Table S4).

Slides were stained immunohistochemically with antibodies against Ki67 according to standard protocols to assess the rate of cell proliferation within tumour sections. Briefly, 2-μm-thick sections from formalin-fixed, paraffin embedded tissue blocks were prepared, deparaffinised and rehydrated. Immunohistochemical staining and detection was performed using an automated Leica Bond 3 machine according to the manufacturer’s protocol. Antibodies raised against Ki67 (Leica, Cat No: NCL-L-Ki67-MM1, 1:100) and ER (Leica, Cat No: NCL-ER-6 F11, 1:500) were used. Stain detection was performed using a bond polymer refine detection kit. Tonsil sections were used as a positive control for Ki67 staining and breast tissue was used for ER staining. Negative controls were processed in the same manner but with the substitution of PBS for the primary antibody. All sections were examined by light microscopy to assess the presence and scoring of expression. The percentage of tumour cells with nuclear expression of Ki67 was estimated. The Allred scoring system was used to assess ER staining.

Methylation data in BCC Tissue Bank tumour biopsies was validated using the TCGA breast tumour for which 450 K Illumina Infinium Beadchip Array data was publically available (n = 365 ER-positive tumours, n = 109 ER-negative tumours). Data was extracted using R software and logistic regression analysis was performed to assess the relationship between ER-status and methylation beta-values at each *ESR1* CpG locus, with histology as an independent variable. The Wilcoxon signed rank sum test was performed with false discovery rate correction as the data was non-parametric.

### Extraction and processing of breast epithelial cell samples from expressed breast milk

Ethics approval for this part of the study was obtained from the Hammersmith Hospital Human Imperial NHS Tissue Bank access committee (reference R13020). Cells were pelleted from frozen 20-ml samples of expressed breast milk in a series of centrifugation and wash steps, and analysed using flow cytometry with a FITC labelled antibody against epithelial membrane antigen (EMA; CD227, Sigma Aldrich; n = 60) and a Cyp5.5-labelled antibody against intracellular ESR1 (Sigma Aldrich; n = 6), according to standard techniques using a FACScalibur flow cytometer (BD Biosciences). DNA was extracted using a phenol:chloroform technique, with duplicate phenol and chloroform steps to optimise yields and reduce phenol contamination, respectively. DNA was bisulphite converted (500 ng) using the EZ-96 Methylation-Gold™ kit (Zymo) and samples from 36 donors were used for hybridisation onto the Infinium HumanMethylation 450 BeadChip array, using the Illumina Infinium HD methylation protocol (conducted by UCL Genomics). The methylation scores from samples on all three chips were processed using standard quality control measures, and normalised (colour correction) and batch adjusted using COMBAT, resulting in beta methylation values according to the fluorescent intensity ratio that ranged from 0 (unmethylated) to 1 (completely methylated). R was also used to analyse the probes related to *ESR1*. Pyrosequencing was subsequently performed for the ESR1 regions described above on 250 ng bisulphite converted DNA samples (n = 53) to validate the *ESR1* regional methylation.

RNA was found to be highly fragmented from frozen milk samples according to assessment by the Bioanalyser 2100 (Agilent Technologies, Santa Clara, CA, USA) using standard techniques, and was unsuitable for further use. A small number of fresh breast milk samples were collected and high quality RNA, according to the results of the Bioanalyser 2100 (RIN score >7), was obtained from 11 samples using a standard Trizol technique. Furthermore, the OD_260/280_ was >1.8 for all 11 samples. cDNA was prepared and qPCR assays for *ESR1* were performed according to the techniques and primers described above for the cell line analysis. EBM samples were normalised against MCF7, and MDA-MB-231 RNA was used as the negative control, along with a negative reverse transcriptase sample.

### Statistical analysis

All experiments were performed in triplicate unless otherwise stated. The mean ± standard deviation (SD) was calculated from each triplicate repeat of the pyrosequencing and qRT-PCR experiments. The mean ± SD were calculated after each replicate, and the standard error of the mean (SE_x_) was then calculated. Parametric data, such as the methylation levels in cells incubated with DMSO and DAC or DMSO alone, were compared using paired t-tests. Non-parametric data, including average methylation levels across the gene body, were compared using unpaired Wilcoxon signed rank sum tests. All statistical tests were two-sided and performed using Microsoft Excel (Microsoft, USA). To validate the expression changes of ESR1 after DAC treatment, two publically available expression datasets were mined for data regarding DAC treatment of two breast cancer cell lines used in this current study (gse10613 and gse13733) [23,24]. The software programmes, R v2.15 and Microsoft Excel, were used to analyse all data.

## Results

### Differences in intragenic methylation (IGM) patterns across ESR1 between ER-pos and ER-neg cell lines

**Figure 1:**
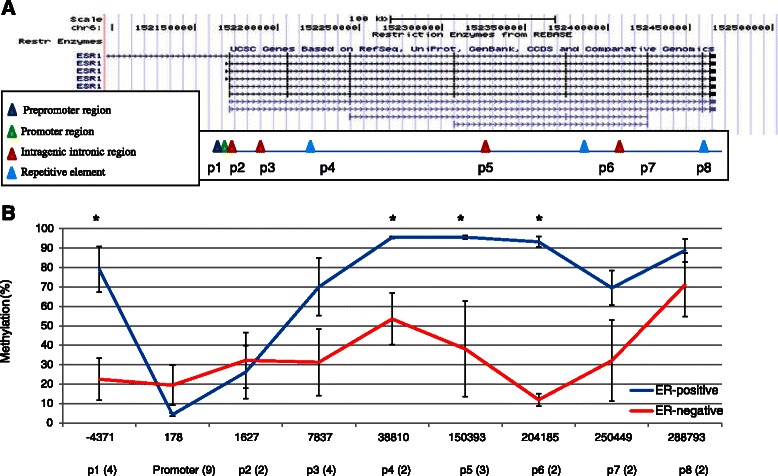
Intragenic DNA methylation in the ESR1 gene. **A)** Schematic showing the position of pyrosequencing assays across the ESR1 gene. **B)** The DNA methylation levels across the *ESR1* gene in three ER-pos cell lines (blue) and three ER-neg cell lines (red). Data was collected in triplicate in each cell line at each locus, and the standard error of the mean was then calculated (error bars); *p < 0.05, Bonferroni corrected t-test.

In total, nine distinct regions across the pre-promoter, promoter and intragenic regions of the *ESR1* gene were assayed by pyrosequencing (Figure 1A). Methylation levels at two and three adjacent CpG loci were averaged for each site, as shown in brackets in the x-axis of Figure 1B, and two distinct patterns were observed (Figure 1B). Pre-promoter methylation levels in ER-positive and ER-negative cells were 79.1% vs. 22.5%, promoter methylation levels were 4.3% vs. 19.5%, and average IGM levels were 80.5% vs. 38.6%, respectively.

ER-positive cells had particularly low levels of methylation at the transcription start site, as would be expected in a transcriptionally active gene. This region of hypomethylation extended into the first intron, with methylation levels of <5% at position 2 in ER-positive cell lines (Figure 1B), 1,627 nucleotides downstream from the TSS. All six cell lines showed increasing methylation levels towards the 3’UTR, regardless of ER expression status.

### In vitro ESR1 methylation changes after DAC treatment

**Figure 2:**
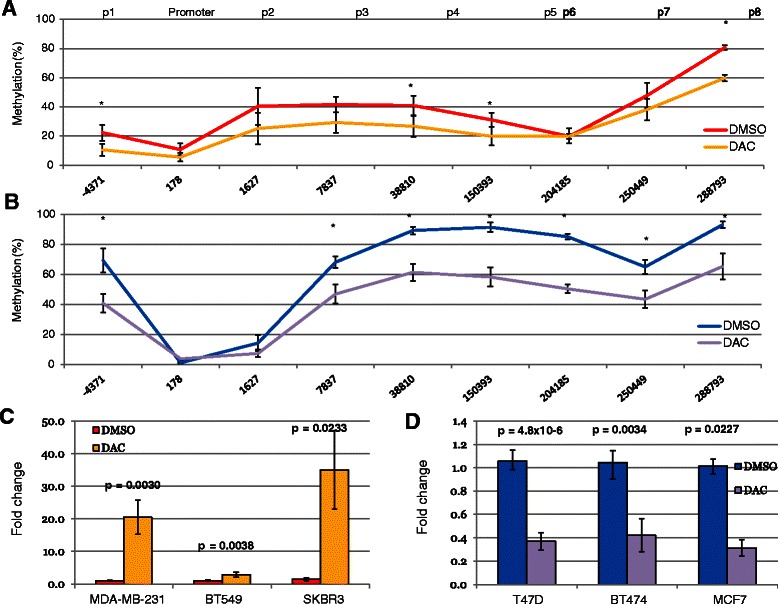
The effect of decitabine on DNA methylation and expression in breast cancer cell lines. **A)** Composite graphs showing the DNA methylation levels across the *ESR1* gene in three ER-neg cell lines treated with DAC (yellow) compared to DMSO-treated controls (red). Data was collected in triplicate in each cell line at each locus, and the standard error of the mean was then calculated (error bars); *p < 0.05, Bonferroni corrected t-test. **B)** DNA methylation across the *ESR1* gene in ER-pos cell lines treated with DAC (purple) compared to untreated (blue). **C, D)** qRT-PCR gene expression in individual cell lines treated with DAC (ER-neg = yellow, ER-pos = purple) compared to DMSO-treated controls (ER-neg = red; ER-pos = blue). Expression increased significantly in ER-neg cell lines, but was significantly reduced in all three ER-pos cell lines (p < 0.05).

After 1 week of treatment with 1 μM DAC, both the ER-negative and ER-positive sets of cell lines showed a consistent decrease in methylation across the *ESR1* gene (P < 0.05; (Figure 2A, B).

The average promoter methylation level after DAC treatment was 3.7% and 5.5% in ER-positive and ER-negative cell lines, respectively, compared to the average post-DAC IGM levels which reduced from 80% to 50% (ER-positive) and 38% down to 31% (ER-negative) (Figure 2A). ER-positive cells had persistently low levels of methylation at the CpG sites in the region of the transcription start site, which extended into the first intron (Figure 2B).

### Expression levels of ESR1 mRNA

We assayed the level of *ESR1* mRNA in all six human breast cancer cell lines in DAC-treated and control cells using RT-PCR. In ER-negative cells, 1 μM DAC increased *ESR1* mRNA expression significantly (MDA-MB-231 cells: 20-fold; p = 0.003; BT549: 3-fold, p = 0.037; SKBR3: 35-fold, p = 0.023; Figure 2C, Additional file 2: Figure S2). In ER-positive cells, treatment with DAC decreased expression ranging from 0.31 in MCF7 cells (p = 0.023) to 0.42 in BT474 cells (p = 0.0034) and 0.37 in T47D (p = 4.8 × 10^−6^; Figure 2D).

We used previously published data to replicate this result using gene expression profiles of MCF7 and MDA-MB-231 cells treated with DAC (gse10613 and gse13733) [23,24]. Expression profiles were categorised into four quartiles, with the highest quartile representing the genes that had the top 25% expression levels prior to DAC treatment. The genes that mirrored the behaviour of ESR1 after DAC treatment in MCF7 cells in our *in vitro* study, could then be identified (Additional file 2: Figure S1, 4th quartile genes with reduced expression after DAC treatment, n = 40). This confirms that ESR1 was one of the most downregulated genes in MCF7 following DAC treatment, and upregulated in MDA-MB-231 s, but also identifies other genes, including several histone proteins, that are similarly downregulated upon DAC treatment.

### ESR1 IGM levels in DNA from breast tumour biopsies

We received 155 breast tumour samples from the Breast Cancer Campaign Tissue Bank. These came in two separate sets: 20 fresh frozen tumour blocks with FFPE slides from the same tumours (ER-positive = 10; ER-negative = 10) and 135 tumours that were provided as formalin-fixed paraffin embedded slides (FFPE; n = 45 ER-neg, n = 45 ER-positive grade 2, n = 45 ER-positive grade 3; 10 slides from each tumour, 3-μm sections).

New primers were designed to accommodate shorter fragments in FFPE derived DNA (Additional file 1: Table S2), and tested in the 20 fresh frozen samples vs. their matched FFPE DNA samples. There was a relatively high correlation between the results gained from the two methods of tissue preparation (r^2^ = 0.77, data not shown), but there was significantly more variation in the FFPE compared to the FF tumours.

**Figure 3:**
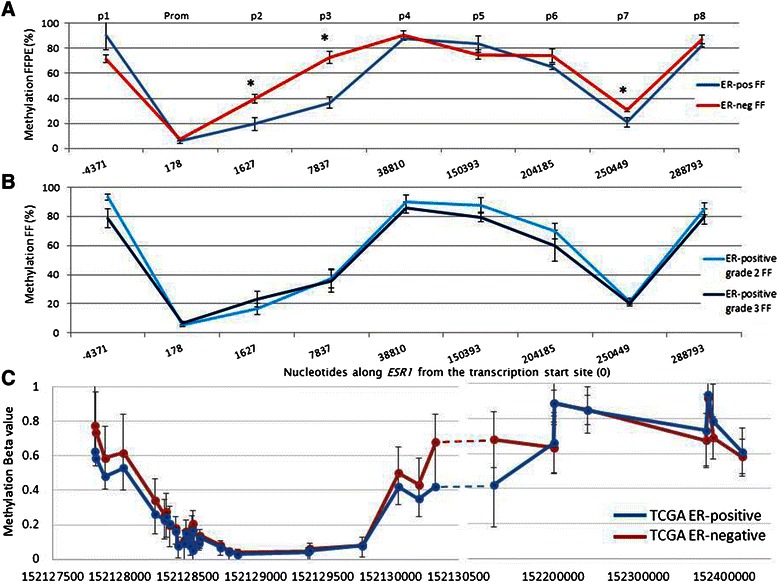
*ESR1* intragenic DNA methylation in breast tumour DNA. **(A)** Pyrosequencing based methylation data across the *ESR1* gene in fresh frozen (FF) human breast tumour samples (n = 10 ER-negative, n = 10 ER-positive [n = 5 grade 2 vs. grade 3]). At regions p2 and p3 (approx. 2–8 kb downstream of the TSS), methylation levels were variable and significantly more methylated in ER-negative tumours, *p < 0.05. **(B)** In ER-positive FF tumours, methylation levels were very similar between grades. **(C)** 450 K Beadchip methylation analysis of *ESR1* methylation from TCGA breast tumours (blue line = ER-positive tumours, n = 365; red line = ER-negative tumours, n = 109). The methylation pattern is very similar to the data shown from breast tissue biopsy material shown in **(A)**, with the only differences appearing within the first intron (equivalent to regions p2 and p3 of the pyrosequenced assays in **(A)**, Wilcoxon rank sum test, *p < 1.0 x 10^−7^.

The promoter region of most tumours was unmethylated regardless of ER status, with average methylation levels of <5% in both ER-positive and ER-negative biopsies. Furthermore, the intragenic pattern of methylation did not show as much variability across the entire gene as was observed in cell lines. The only region that was significantly different between ER-positive and ER-negative tumours were the two CpG sites 7,837 bp from the transcription start site in the first intron (MVR p3; p = 0.01). However, in contrast to the cell lines, the methylation levels at this region were higher in the ER-negative FF tumours (82.1%) compared to ER positive tumours (57.3%; Figure 3A), which was not predicted by the *in vitro* studies. This site was not differentially methylated between grade 2 and 3 ER-positive tumours, although grade 2 tumours were slightly higher methylated at this region than grade 3 (62.1% vs. 51.5%; p > 0.05; Figure 3B).

A potential explanation for the difference between cell lines and tumour data may relate to cell proliferation rates, which also correlate with intragenic DNA methylation [25]. An analysis of cell proliferation was performed by staining human cancer tumour sections (n = 45 ER-pos, n = 45 ER neg) with an antibody against Ki67, which identifies the degree of cell proliferation. As expected, ER-negative tumours had significantly higher levels of Ki67 staining, and therefore higher proliferation, than ER-positive tumours (Table 1). The higher number of cells that are actively proliferating (i.e., during the S phase of the cell cycle; data not shown), may therefore, have higher IGM levels, and provide an explanation for the higher levels of IGM observed in the ER-negative tumours. The degree of Ki67 staining was positively correlated with increased methylation levels at p2, p3 and p7, which were also the regions that were differentially methylated between ER-negative and ER-positive breast cancers (data not shown).

**Table 1:** **Ki67 scores of stained tumour sections**

Ki67 score	Mean (%)	t-test	MVR p3 region	Mean (%)	t-test
ER-pos	10.00	0.0004	ER-pos	57.3	0.01
ER-neg	68.11		ER-neg	82.1	

Data from the TCGA dataset showed a markedly similar pattern of methylation across *ESR1* to those of the BCC Tissue Bank tumour samples described above (Figure 3C). In particular, ER-negative tumours showed significantly higher beta methylation values than ER-positive tumours at the three cg loci located within the first intron (cg04063345, cg15626350, cg00601836; p < 1.0 × 10^−7^ for all), which were in the same region as the p2 MVR assayed by pyrosequencing.

### Methylation levels in *ESR1* in breast epithelial cells from EBM samples

**Figure 4:**
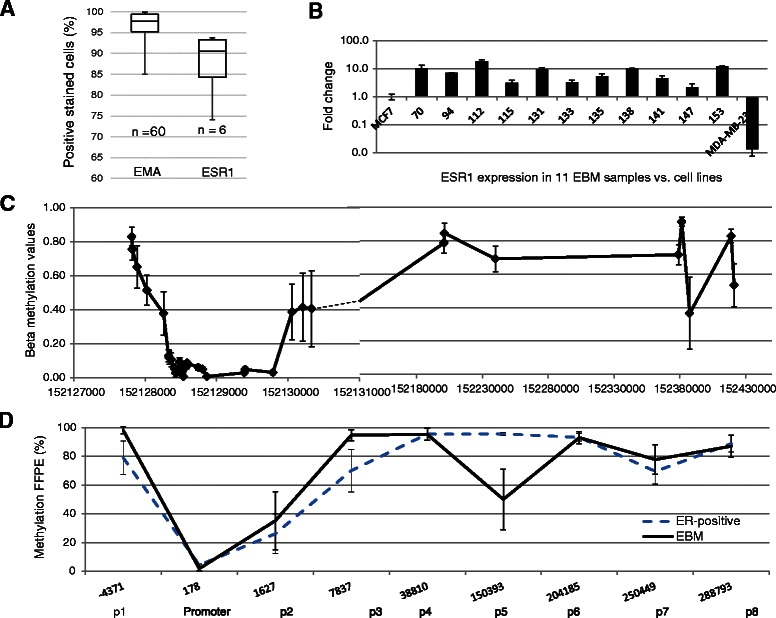
Intragenic DNA methylation in ESR1 in exfoliated breast epithelial cells from expressed breast milk (EBM). **(A)** Flow cytometry of epithelial membrane antigen (EMA; median positive staining, 97.8%; 95.3%-99.4%; n = 60) and intracellular ESR1 (median, 90.5%; IQR, 84.4%-93.3%; n = 6). **(B)** qRT-PCR analysis of 11 EBM epithelial cell RNA samples showing high levels of ESR1 expression normalised against ER-pos MCF7 and ER-neg MDA-MB-231 cell lines, (range 2.2- 19-fold higher). **(C)** Methylation values from 450 K analysis of 36 DNA samples extracted from breast epithelial cells from expressed breast milk. The overall pattern matched those of ER-positive cancer cell lines, and regions of variability were found at regions that mapped to p2 and p7. **(D)** Validation of the methylation levels using pyrosequencing in a separate set of 53 EBM samples (black solid line). The pattern of methylation is very similar to that of the 450 K analysis of EBM samples and of ER-pos cancer cell lines (dashed line) with high levels of variability at p2 for both.

Initial flow cytometry studies indicated that the median percentage of epithelial cells within the cell pellet from expressed breast milk was 97.8% (n = 60; IQR, 95.3%-99.4%; Figure 4A). The proportion of stem cells was less than 1% (CD042 antibody staining, data not shown). *ESR1* expression has not been characterised previously in breast epithelial cells from human expressed breast milk. Flow cytometric analyses in a small number of EBM samples (n = 6) was conducted to assess the presence of intracellular ESR1, and the median percentage of positive cells was 90.5% (IQR, 84.4%-93.3%; Figure 4A). ESR1 expression was confirmed by qRT-PCR assays for *ESR1* mRNA expression in 11 samples of freshly expressed breast milk and was expressed ~10-fold higher than MCF7 (Figure 4B).

We profiled DNA methylation in 36 individuals using the Illumina Infinium HumanMethylation 450 BeadChip and all samples passed the quality control procedures. Data from the *ESR1*-associated 450 K probes was extracted and showed low levels of promoter methylation (Figure 4C). Pyrosequencing of a separate set of bisulphite converted DNA samples (n = 53) validated the 450 K ESR1 methylation profiles (Figure 4D). Methylation levels rose rapidly within the first intron, but was highly variable between samples as evidenced by the wide error bars in both the 450 K data and at the region analogous to p2 in the pyrosequencing assays (Figure 4C, D). IGM levels remained high at most regions assayed along the *ESR1* gene, in a pattern that was very similar to that shown for ER-positive tumour cell lines (Figures 1B and 4C, D). Very high levels of *ESR1* mRNA expression were found in all 11 samples, however, we found no significant correlation with the variable methylation in this small subset of samples (r^2^ = 0.0082).

## Discussion

This study identified marked and reproducible differences in the pattern of IGM across *ESR1 in vitro* in ER-positive compared to ER-negative cell lines, which was supported by similar methylation patterns in the strongly ER-positive breast epithelial cells from breast milk samples. Promoter regions were uniformly methylated in ER-negative cell lines, and unmethylated in ER-positive cells. As predicted, demethylation with DAC treatment increased the transcription of ESR1 in all three ER-negative cell lines, but the most surprising finding from this study was that DAC resulted in decreased levels of expression in ER-positive cell lines, via a mechanism independent from promoter methylation. Of note, the patterns of promoter and IGM established in tumour cell lines and a homogenous population of breast epithelial cells from EBM samples were highly similar, but differed markedly from those generated from two sources of ER-positive or ER-negative tumour biopsy samples (BCC Tissue Bank and the TCGA database). These observations are likely to reflect various caveats, including cell type heterogeneity and the tissue microenvironment, which results in a mixed epigenetic signal in tissue samples. This finding was in contrast to the artificial nature of cell lines grown on plastic in the presence of high concentrations of growth factors and has important implications on the choice of tissue for epigenetic analyses.

The principal focus to date of the transcriptional effects of DNA methylation has been on promoter-associated CpG islands. The results of this current study were in accordance with recent findings by Yang et al., which indicated that DAC treatment reduced the expression levels of overexpressed genes [26]. The functional mechanism by which IGM exerts a transcriptional effect remains unknown, but the transcriptional changes observed for ESR1 in ER-positive cells may represent a therapeutic target. With the advent of array techniques that examine greater proportions of the genome, including high-density microarrays and next generation sequencing based DNA methylation analyses, the functional roles of IGM are becoming more apparent [27] and are linked to gene expression [17,18,28]. IGM levels have been shown to change markedly during carcinogenesis [27], but in the absence of precise roles in the normal state, the effects of disrupted IGM levels in aberrant cells cannot be predicted or quantified. Several mechanisms by which IGM may be functionally important have been proposed, including the prevention of transcription from alternative start sites in the gene body, chromatin regulation, the inhibition of transposable elements, and the control of alternative splicing [29,30]. Furthermore, high IGM levels may prevent the transcription of non-coding RNA in the antisense direction, although this finding was not supported by the results of this current study. Moreover, the methylation of intragenic transposable elements may affect transcription efficiency by impeding RNA polymerase II along the gene body [31], although recent evidence suggests that intragenic DNA methylation represents a by-product of the chromatin assemblies related to transcription, and has no direct impact on transcription efficiency [8].

DAC is currently used as a clinical treatment for myelodysplastic syndrome and acute myeloid leukemia [32]. DAC is incorporated into double-stranded DNA during cell replication, and therefore more rapidly dividing cells might show greater levels of demethylation. The ER-positive breast cancer cell lines in this study were passaged more frequently than ER-negative ones, and all cell lines were cultured in media that contained oestrogen, fuelling a higher rate of replication in the ER-positive cells. A link between DNA methylation and proliferation has previously been proposed by our group and others [19,33]. Aran et al. observed that proliferating cells and tissues tended to have higher levels of IGM [19]. This observation was corroborated by our Ki67 and methylation data in tumour biopsies where the ER-negative tumours had a higher proportion of proliferating cells than the ER positive tumours (Table 1). This leads us to hypothesise that intragenic methylation levels may be influenced by the cell proliferation rate. In terms of the DNA from tumours biopsies, ER-negative tumours had a much faster rate of cell proliferation, as indicated by the significantly higher levels of staining with Ki67 compared to ER-positive cells (Table 1). It is likely that the higher levels of IGM observed in ER-negative tumours were influenced by the higher levels of cell proliferation in these tumours. Velicescu et al. noted that serum starvation stalls cells at the G0/G1 phase of the cell cycle, preventing cell division and that DNA methyltransferases were predominantly expressed during the S phase [34]. Active demethylation of promoter regions is known to be initiated by the same enzymes that induce methylation (DNMT3A and 3B) [35,36], but is a long and energy expensive process [37]. To establish if cell proliferation does actively affect IGM levels, and the subsequent effects on transcription, larger studies that investigate different cell lines and clinical samples with a genome-wide microarray approach may be required.

The genomes of cancer cells undergo massive epigenetic changes with the loss and redistribution of methylation. During neoplastic change, the CGI-associated promoter regions of multiple genes across the genome become focally hypermethylated [3,38,39], which may occur concurrently with genome-wide hypomethylation in tumour cells from a variety of cancers, including breast cancer [40,41]. These IGM changes may follow a distinct order during carcinogenesis and provide biomarkers of breast cancer risk in healthy women [42,43], as has already been proposed in ovarian cancer [44]. However, the pathological mechanisms and implications of genome-wide demethylation are not understood, but may result in the reactivation of repetitive elements that are usually hypermethylated, with consequent genomic instability [45].

The observed intragenic *ESR1* methylation in epithelial cells extracted from human breast milk confirmed the *in vitro* cell line findings of high levels of IGM in ER-positive tumour cell lines, and low levels of promoter methylation. This suggested that if a homogenous cell type is investigated, the epigenetic profile is also more homogenous compared to the tumour biopsy material, where the cell-specific signatures create a mixed signal [46]. From the limited number of samples of RNA available from EBM (n = 11), no correlation was found between the highly variable methylated region at p2 in *ESR1* and ESR1 expression, however, further studies in a larger number of samples will be required to investigate such associations.

Of note, this is the first study to show the feasibility of using DNA extracted from cells in EBM for 450 K DNA methylation analyses. All 36 samples passed the quality control procedures, with a relatively low level of excluded probes. Although the DNA from such frozen milk samples is relatively fragmented (data not shown), sufficient quality is retained to enable both array and PCR-based assays to be performed. Given that these cells are in a highly proliferative state during lactation [47], and represent the most oestrogen responsive breast epithelial cell type, it will be important to collect and characterise further samples of these cells in the future to gain a greater understanding of the normal biology of ductal epithelial cells, and how their epigenetic status differs from cancer cells [48]. They also represent an important resource in which the impact of environmental and intrinsic cancer risk factors can be assessed.

### Limitations

This study had several limitations. Firstly, we have not investigated the effect of passive demethylation via decitabine on distant enhancers or the many alternate promoters of *ESR1*. Secondly, the results from breast tumour biopsies could have been confounded by the presence of 5’hydroxymethylation, which is present in primary tissue but not in *in vitro* cultured cell lines (and high passage number cell lines in particular). Future studies that use novel techniques such as oxidative bisulphite sequencing for the detection of this epigenetic mark will be needed to assess this. Thirdly, the quality of DNA extracted from FFPE tumour sections was relatively poor compared to that of fresh frozen samples, leading to higher technical variation which may have further confounded the analysis. The technical variation in gene expression may have been reduced by using alternative control genes to GAPDH [49]. Finally, the number of samples of epithelial cells from EBM was relatively small, particularly for freshly expressed samples from which it was possible to extract RNA for further assays and larger studies are warranted. In accordance with most previous reports, this study demonstrated a correlation between methylation and expression levels. While it is possible to remove methylation with decitabine and show the reciprocal changes in expression, we have not shown the reverse of adding intragenic methylation to a gene and showing a reciprocal increase in expression. Only now, with advances in the use of CRISPR technology, is there promise that such an experiment might be possible, and future investigations will examine whether this technique can work reliably for DNA methylation studies [50].

## Conclusions

Numerous studies that have investigated demethylating agents have shown that genes can be reactivated by the demethylation of promoter-associated CGIs. However, the repression of gene transcription by demethylation is a more recent discovery. In this study, the reduction of ESR1 transcription after DAC treatment in ER-positive cells was investigated further in the search for functional insights into IGM. This study adds to our understanding of the methylation status of intragenic CpG sites, and may provide a mechanism for the down-regulation of ESR1 expression.

## Additional files

Additional file 1: Table S1. Primer sets for nested, semi-nested and single round PCR, in addition to sequencing primers for pyrosequencing. The chromosomal coordinates denote the CpG sites on chromosome 6 analysed by pyrosequencing. Some loci were amplified using two sets of primers to avoid repetitive elements. **Table S2.** Primers for RT-PCR assays. **Table S3.** RT-PCR primers designed to amplify MVR regions of *ESR1*. **Table S4.** Additional primers designed to amplify regions of interest across *ESR1* in human biopsy material.
Additional file 2: Figure S1. The heatmap shows the relative protein expression of very highly expressed genes in MCF7 cells (n = 40 genes) that are downregulated after DAC treatment, compared to DAC-treated MDA-MB-231 cells from two publically available datasets (gse10613 and gse13733). Pink indicates upregulation, while blue indicates downregulation. The changes in ESR1 expression levels are in accordance with the findings from our current *in vitro* studies. **Figure S2.** Average delta-CT values for each cell line (control vs. DAC-treated), which demonstrates that DAC treatment caused significant increases in expression in ER-neg cell lines (SKBR3, BT549, and MDA-MB-231).
