# Supplementary material for: Transcriptional implications of intragenic DNA methylation in the oestrogen receptor alpha gene in breast cancer cells and tissues
Source: BMC Cancer. 2015 May 1;15:337. doi: 10.1186/s12885-015-1335-5 (PMC4424887; doi:10.1186/s12885-015-1335-5)
Supplement: Additional file 1: Table S1. — Primer sets for nested, semi-nested and single round PCR, in addition to sequencing primers for pyrosequencing. The chromosomal coordinates denote the CpG sites on chromosome 6 analysed by pyrosequencing. Some loci were amplified using two sets of primers to avoid repetitive elements. Table S2. Primers for RT-PCR assays. Table S3. RT-PCR primers designed to amplify MVR regions of ESR1. Table S4. Additional primers designed to amplify regions of interest across ESR1 in human biopsy material. [file 12885_2015_1335_MOESM1_ESM.doc]

**Additional file 1**

**Table S1:** Primer sets for nested, semi-nested and single round PCR, in addition to sequencing primers for pyrosequencing. The chromosomal coordinates denote the CpG sites on chromosome 6 analysed by pyrosequencing. Some loci were amplified using two sets of primers to avoid repetitive elements.

√, biotin tag added; Rev, reverse (3’ – 5’) sequence primer.

| **Site** | **Chr location of CpG sites** | **Primer** | **Biotin** | **Primer sequence** |
| --- | --- | --- | --- | --- |
| p1a | 152165538 | F1 |  | GTTATGTTTGGTTATAGTATTATTTGTTTG |
|  | 152165542 | R1 |  | CACCACACCCAACTAATTTTTTT |
|  |  | F2 |  | GGTTTTAGTTGGTATGAAGATATAAAGA |
|  |  | R2 | √ | GACGGGACACCGCTGATCATCGTTTACTAATCCTAAACTCCTAACCACAAATAAT |
|  |  | S1 |  | TTTAGTAAAAATAGTTTTGAAAGA |
|  |  |  |  |  |
| p1b | 152166475 | F3 |  | GTTGGGTTATGTTAAGATGGATTTT |
|  | 152166508 | R3 |  | CTTTACAATCTCTCTTTTTCCATTACAT |
|  |  | F4 |  | GTAAATAGGTTGTAGGGTTTAGGATGAA |
|  |  | R4 | √ | GACGGGACACCGCTGATCGTTTATAAAACCCATTACCTTACCTATCC |
|  |  | S2 |  | GGGTTTAGGATGAAGTT |
|  |  |  |  |  |
| p2 | 152171981 | F1 |  | GGAGTTAGTTGGTTGAGT |
|  | 152171995 | R1 |  | ATTAAAATAAAAACACACAACCTTC |
|  |  | F2 |  | GATTGTTGGTATGTGATTTTTGATAG |
|  |  | R2 | √ | GACGGGACACCGCTGATCGTTTAAAAATAATCTCCTATATCCTAAACC |
|  |  | S1 |  | GTTTAGTGATAAGTTTGTTTTATT |
|  |  |  |  |  |
| p3a | 152177914 | F1 |  | GTAAATTTGGTTTTAGGAGTTTTAGATT |
|  | 152177934 | R1 |  | TCCCTATAACTAAAAAAAATAACTTCTCAA |
|  |  | F2 | √ | GACGGGACACCGCTGATCGTTTAAATTATATTATAAAGGTATTTTTTTAATGGT |
|  |  | R2 |  | ACCCTTTCACTCTAAATTACTTAAAA |
|  |  | S1 | Rev | CTATATTTCTAACAATATTACTTTC |
|  |  |  |  |  |
| p3b | 152178481 | F3 |  | GGAAGGGATTTTTAGATGTTATTGG |
|  | 152178518 | R3 |  | ACCATTTTAAACAACAAAATCATCAAC |
|  |  | F4 |  | AGAATGTGGGAAGTTTGTGAA |
|  |  | R4 | √ | GACGGGACACCGCTGATCGTTTAACCTTACTCAACCTCAACTAAAAACATAT |
|  |  | S2 |  | TTATGGATTTTGTATTTGTAAA |
|  |  |  |  |  |
| p4a | 152209019 | F1 |  | GAGTTAAGGGAGTATGGTGAAGAA |
|  |  | R1 |  | ATATTTCCCTTTTATACACACAAACA |
|  |  | F2 |  | AGTGAAGATTAGGAGGAAAGGTAGA |
|  |  | R2 | √ | GACGGGACACCGCTGATCGTTTAATCCATAACCCAAAAATATCTTTATTTAAC |
|  |  | S1 |  | GGAGGAAAGGTAGAG |
|  |  |  |  |  |
| p4b | 152209337 | F3 |  | TTTGGAATTAAAAAGTTTTTGTTGAAATTG |
|  |  | R3 |  | CACTAAAAACCATTACTCAATCTCT |
|  |  | F4 |  | AAGGATGGTGAAGGGTTTGAT |
|  |  | R4 | √ | GACGGGACACCGCTGATCGTTTACCTCTTCAACCAAAACACTTAAAACATA |
|  |  | S2 | Rev | ATGGTGAAGGGTTTGATA |
|  |  |  |  |  |
| p5a | 152320752 | F1 |  | AGATGTGATTGTTTTAGGATTTGAATTAA |
|  | 152320791 | R1 |  | CCAAATACAATTACACAACTTAAAAACTAT |
|  |  | F2 |  | GGTAATTTTATAGTTAAAGGGATTTAAGTT |
|  |  | R2 | √ | GACGGGACACCGATGATCGTTTAACTCCAATAACCCCATTAAACC |
|  |  | S1 |  | AGGGATTTAAGTTTATTTGAT |
|  |  | S1b |  | GGATAGGTTTATATTTTTAAGGT |
|  |  |  |  |  |
| p5b | 152321053 | F3 |  | ATTTATATTAAGGTTTTTTTGGGGAAA |
|  |  | R3 |  | ACCACATCCTACAATACTTTCTAAAT |
|  |  | F4 |  | ATGAGTTAGGGATTATTTTTGTGGATAT |
|  |  | R4 | √ | GACGGGACACCGCTGATCGTTTAAACACACAAAAACCACACTACC |
|  |  | S2 |  | GTATTGAGATGATAATTAGTTTGA |
|  |  |  |  |  |
| p6 | 152375048 | F1 |  | ATTTGAGTTAGTAGGGTTTTTTTTGTT |
|  | 152375095 | R1 |  | TACTCCTAAACTACAACCAAATCACTTACC |
|  |  | F2 |  | TTATTTTTTAGAATGTGTTTGGTTAGAGAT |
|  |  | R2 | √ | GACGGGACACCGCTGATCGTTTAACAATAACTTCCCTAAATACTCCA |
|  |  | S1 |  | AGAGATTTTGATGATTGGT |
|  |  |  |  |  |
| p7 | 152420889 | F3 |  | GGGGAAAATGTTTGGAGGATGAA |
|  | 152420918 | R3 | √ | gacGGGACACCGCTGATCGTTTATAAACACAAACTCCCTCCCACACT |
|  |  | S2 |  | GAGAAATGTTTTTTTTTTAGTATAA |
|  |  |  |  |  |
| p8 | 152459154 | F3 |  | TTGGGTTTATATTAGGATTTTAGGAATG |
|  | 152459188 | R3 |  | TTCCCTCATTTCTTTATTCACTCAC |
|  |  | F4 |  | GGTTATGAGGGAAGGTTTATGA |
|  |  | R4 | √ | GACGGGACACCGCTGATCGTTTAAATACATCTATTTTCCCAAAAACTAA |
|  |  | S2 |  | TTTATGATGTTTTAAGGAGG |
|  |  |  |  |  |

**Table S2:** Primers for RT-PCR assays.

| **Gene** | **Primer** | **Primer sequence** |
| --- | --- | --- |
| **ESR1** | Forward | GGTGCCTGAGACACAGACC |
| **ESR1** | Reverse | GTGAGAGAACAGAAACTGGC |
| **GAPDH** | Forward | TCCCTGAGCTGAACGGGAAG |
| **GAPDH** | Reverse | GGAGGAGTGGGTGTCGCTGT |

**Table S3:** RT-PCR primers designed to amplify MVR regions of *ESR1*.

| **Site** | **Direction** | **Primer sequence** |
| --- | --- | --- |
| **P2 (intron 1) sense** | Forward | CCAGGACACAGGAGACCACT |
| **P2 (intron 1) sense** | Reverse | TCTCCTCTCCCTCCCATTTT |
|  |  |  |
| **P3 (intron 1) sense** | Forward | TTTGTTTTCCCAGCAGTTCA |
| **P3 (intron 1) sense** | Reverse | ACCAGAATCTCCTGCATGTG |
|  |  |  |
| **P4 (intron 2) sense** | Forward | **G**CAGCTCTCCCTGTTTTCCT |
| **P4 (intron 2) sense** | Reverse | AAAGTGTTTGGCTTGAAAGCA |
|  |  |  |
| **Exon 2 sense** | Forward | TTGGCCAGTACCAATGACAA |
| **Exon 2 sense** | Reverse | TGCTTCAGGCTACCATTATGG |
|  |  |  |
| **Exon 2 sense** | Forward | CCATAATGGTAGCCTGAAGCA |
| **Exon 2 sense** | Reverse | TTGTCATTGGTACTGGCCAA |
|  |  |  |

**Table S4:** Additional primers designed to amplify regions of interest across *ESR1* in human biopsy material.

| **Site** | **Primer** | **Primer sequence** |
| --- | --- | --- |
| P1 | F1 | ATTTTGTTTGGTTGAAGTATAGGT |
| P1 | R1 | gacGGGACACCGCTGATCGTTTACAAATCATAACTTCCCCCATAAATTCA |
| P1 | S1 | TTTGGTTGAAGTATAGGTT |
| Prom1 | F1 | GGGTTGTGTTTTTTTTTTAGGTG |
| Prom1 | R1 | gacGGGACACCGCTGATCGTTTAACCATCCCAAATACTTTAATATAAAAAAT |
| Prom1 | S1 | GGGGATAAGGTTTGTATT |
| Prom 2 | F1 | AGTATTTGGGATGGTTTTATTGTATTAGA |
| Prom 2 | R1 | gacGGGACACCGCTGATCGTTTAACTTACTACTATCCAAATACACCT |
| Prom 2 | S1 | GGATGGTTTTATTGTATTAGAT |
| Prom 3 | F1 | GTTTGGGGGGTTTTTTTTTATTTAA |
| Prom 3 | R2 | gacGGGACACCGCTGATCGTTTATCTCCAAATAATAAAACACCTACTAAC |
| Prom 3 | S1 | GGGGGGTTTTTTTTTATTTAAT |
| P2 | F1 | GGTGAATATTTTTTTAGGTGGTTATAGGAT |
|  | R1 | gacGGGACACCGCTGATCGTTTAACCTAATTCAAAACCCAACCAT |
|  | S1 | GGTTATAGGATAGGTGTATT |
| P3 | F1 | AGGAATTTTTGAGTATTGGTTTAAATAGAA |
|  | R1 | gacGGGACACCGCTGATCGTTTAACTAAAACAAACCATATCCCTAATACTT |
|  | S1 | AGTTTTTAGTAAAGTGTATGGTA |
| P4 | F1 | TTTAAGGATGGTGAAGGGTTTGAT |
|  | R1 | gacGGGACACCGCTGATCGTTTAACCAAAACACTTAAAACATAAAAATAATCT |
|  | S1 | ATGGTGAAGGGTTTGATA |
| P5 | F1 | AGTTTTTATATTGAAGGGAAATTATTTGT |
|  | R1 | gacGGGACACCGCTGATCGTTTACTATTCAATATCTATTCACCACTTATACTC |
|  | S1 | ATATTGAAGGGAAATTATTTGTA |
| P6 | F1 | TTTTTAGAATGTGTTTGGTTAGAGAT |
|  | R1 | gacGGGACACCGCTGATCGTTTAACAATAACTTCCCTAAATACTCCA |
|  | S1 | AGAGATTTTGATGATTGGT |
| P7 | F1 | TTGGTTAGGAATAATAGGATATTAGTGAGA |
|  | R1 | gacGGGACACCGCTGATCGTTTAACTTAACAATTCTCCTTTCTTCTTATC |
|  | S1 | ATTTTTGTTGGAAGAGT |
| P8 | F1 | AGGGTTATAGGGTAAGTGTTAGA |
|  | R1 | gacGGGACACCGCTGATCGTTTACTACCCCAAAACACATACTTTAAAC |
|  | S1 | AGTGTTAGAGAAGGTATAGA |
