# Supplementary material for: Transcriptional implications of intragenic DNA methylation in the oestrogen receptor alpha gene in breast cancer cells and tissues
Source: BMC Cancer. 2015 May 1;15:337. doi: 10.1186/s12885-015-1335-5 (PMC4424887; doi:10.1186/s12885-015-1335-5)
Supplement: Additional file 2: Figure S1. — The heatmap shows the relative protein expression of very highly expressed genes in MCF7 cells (n = 40 genes) that are downregulated after DAC treatment, compared to DAC-treated MDA-MB-231 cells from two publically available datasets (gse10613 and gse13733). Pink indicates upregulation, while blue indicates downregulation. The changes in ESR1 expression levels are in accordance with the findings from our current in vitro studies. Figure S2. Average delta-CT values for each cell line (control vs. DAC-treated), which demonstrates that DAC treatment caused significant increases in expression in ER-neg cell lines (SKBR3, BT549, and MDA-MB-231). [file 12885_2015_1335_MOESM2_ESM.pptx]

## Slide 1
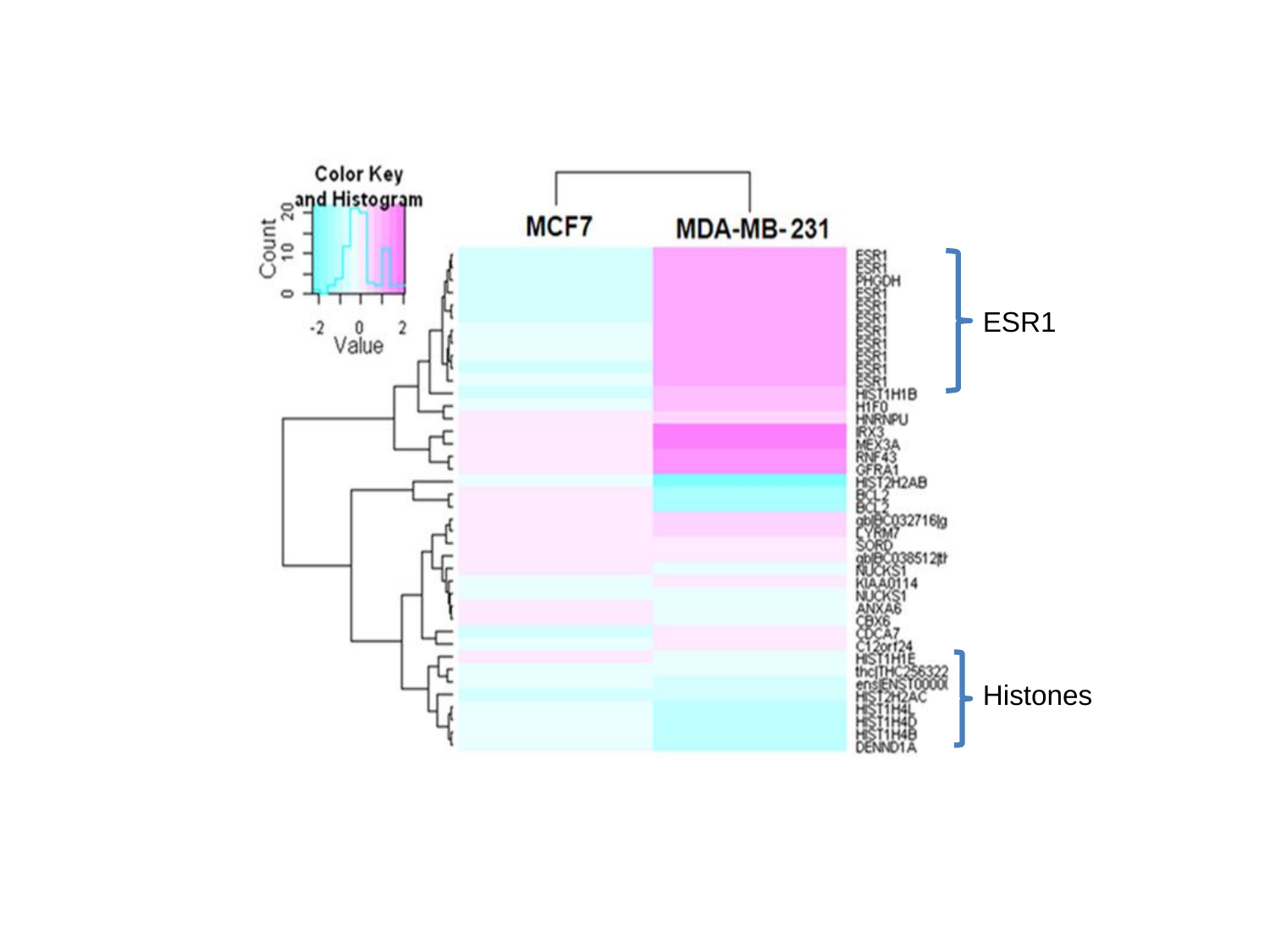

ESR1
Histones

## Slide 2
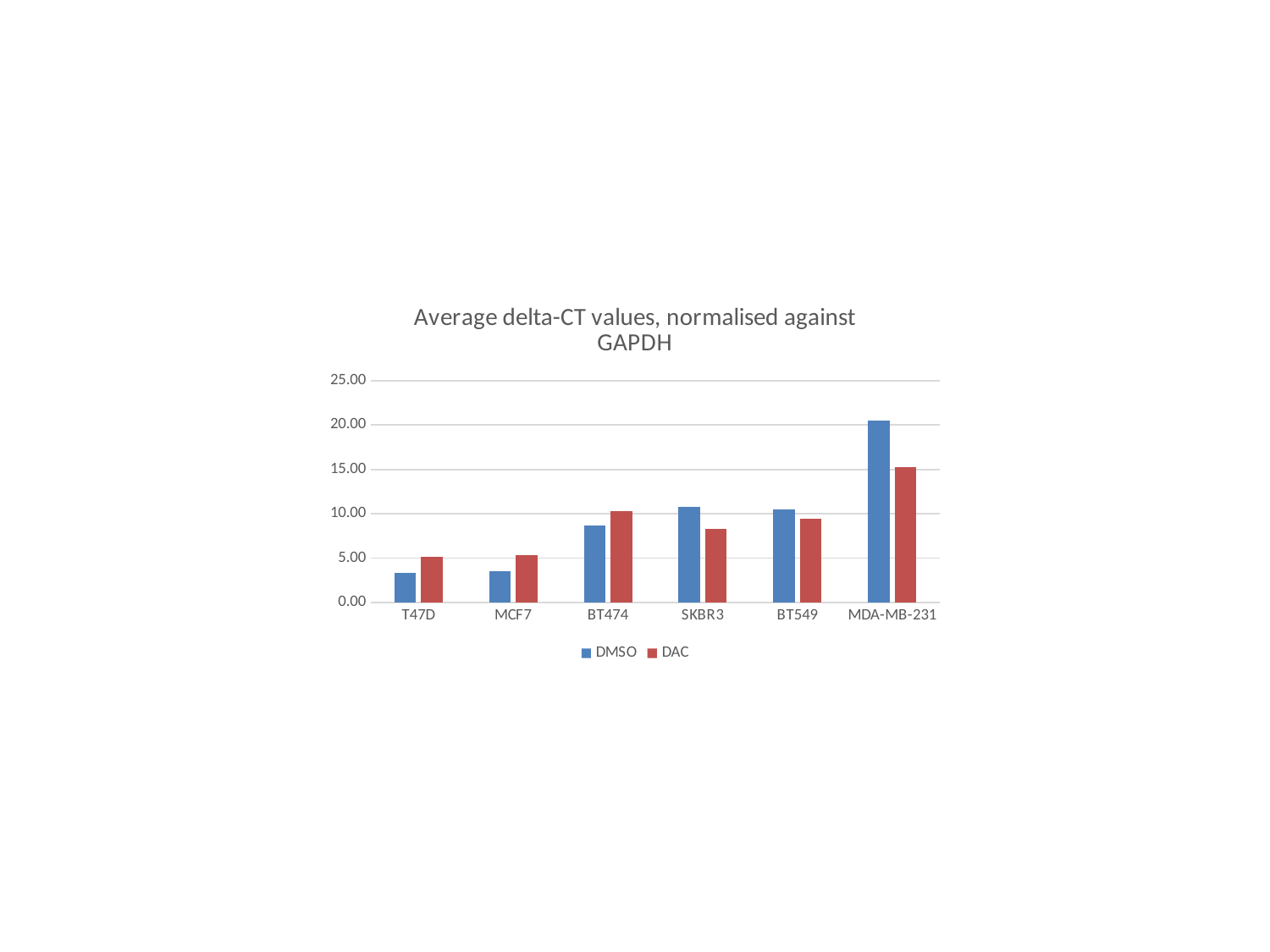

### Chart: Average delta-CT values, normalised against GAPDH
| Category | DMSO | DAC |
|---|---|---|
| T47D | 3.3 | 5.1499999999999995 |
| MCF7 | 3.552283664741864 | 5.304451415367517 |
| BT474 | 8.636902126902333 | 10.30016428355304 |
| SKBR3 | 10.811514034010424 | 8.290945587327444 |
| BT549 | 10.484413549495248 | 9.462486487403407 |
| MDA-MB-231 | 20.469074493915002 | 15.221487244068543 |
